# Alternative *CHRNB4* 3′-UTRs Mediate the Allelic Effects of SNP rs1948 on Gene Expression

**DOI:** 10.1371/journal.pone.0063699

**Published:** 2013-05-14

**Authors:** Xavier Gallego, Ryan J. Cox, James R. Laughlin, Jerry A. Stitzel, Marissa A Ehringer

**Affiliations:** 1 Institute for Behavioral Genetics, University of Colorado Boulder, Boulder, Colorado, United States of America; 2 Department of Integrative Physiology, University of Colorado Boulder, Boulder, Colorado, United States of America; NIGMS, NIH, United States of America

## Abstract

Common genetic factors strongly contribute to both nicotine, the main addictive component of tobacco, and alcohol use. Several lines of evidence suggest nicotinic acetylcholine receptors as common sites of action for nicotine and alcohol. Specifically, rs1948, a single-nucleotide polymorphism (SNP) located in the *CHRNB4* 3′*-*untranslated region (UTR), has been associated to early age of initiation for both alcohol and tobacco use. To determine the allelic effects of rs1948 on gene expression, two rs1948-containing sequences of different lengths corresponding to the *CHRNB4* 3′*-*UTR were cloned into pGL3-promoter luciferase reporter vectors. Data obtained showed that the allelic effects of SNP rs1948 on luciferase expression are mediated by the length and species of transcripts generated. In addition, it was found that miR-3157 increased the overall luciferase expression while miR-138, a microRNA known to play a role in neuroadaptation to drug abuse, decreased luciferase expression when compared to basal conditions. These findings demonstrate the importance of SNP rs1948 on the regulation of *CHRNB4* expression and provide the first evidence of CHRNB4 down-regulation by miR-138.

## Introduction

Tobacco, specifically nicotine, and alcohol are often co-abused substances. Several lines of evidence suggest shared biological and genetic mechanisms [1], [2]. Data obtained from genetic studies utilizing twins and families support the idea that there is a strong contribution from common genetic factors [3], [4], [5]. Furthermore, Genome Wide Association Studies (GWAS) have provided evidence that genetic variants in nicotinic acetylcholine receptor (nAChR) subunit genes (*CHRN* genes) are associated with nicotine dependence [6], [7], [8]. Some of the markers from these studies also show an association with several intermediate phenotypes for alcohol use disorders [9], [10]. These findings suggest that nAChRs may be a common site of action for nicotine and alcohol.

Neuronal nAChRs are ligand-gated ion channels composed of five subunits. To date, eight alpha (α2-α7 and α9-α10) and three beta (β2-β4) subunits have been localized in the mammalian nervous system. The pharmacological and functional properties of each nAChR subtype vary depending on their subunit composition and stoichiometry [11], [12]. Many studies have shown that the stoichiometry of nAChRs, which determines their functional properties, depends on the ratio of available subunits [13], [14], [15], [16].

Taking into account that most of the *CHRN* variants associated with nicotine and alcohol dependence are located in non-coding regions (NCRs), these single nucleotide polymorphisms (SNPs) might alter the expression of the affected subunit and lead to changes in the functionality of nAChRs. The strongest genetic contribution to nicotine dependence comes from variations in the chromosome 15q25 region [17], [18], [19], [20], [21], [22], [23], [24], [25], [26], [27], [28], [29]. Some of these variants have been independently shown to contribute to the occurrence of alcohol use [9], [30], [31]. This region contains the α5, α3, and β4 nAChR subunit gene cluster (*CHRNA5/A3/B4*), whose overexpression in mice has been shown to modify the reinforcing effects of nicotine [32] and ethanol intake [33]. In addition, deletion of the α5 or overexpression of the β4 nAChR subunits in mice has been shown to modulate the aversive properties of nicotine [34], [35]. These studies have highlighted the importance of the balanced and spatial expression of β4 and α5 nAChR subunits on nicotine-addictive behaviors, therefore making it necessary to further understand the elements that coordinate the regulation of this cluster of genes in humans.

Although the transcriptional regulation of the *CHRNA5/A3/B4* cluster of genes has been extensively studied in rats by several groups [36], [37], [38], [39], [40], [41], [42], [43], [44], [45], [46], [47], [48], [49], [50], little is known about the impact of human non-coding SNPs in this cluster of genes on the expression of these subunits. Only few studies describing the functional features of the α3 nAChR [51], [52], [53], [54] and α5 nAChR [55] subunit promoters have been done using human sequences, but none of them have looked at rs1948, located in the 3′-untranslated region (3′-UTR) of *CHRNB4*. To study the effects of rs1948 on the expression of this cluster of genes we have generated constructs placing fragments of the genomic sequence containing rs1948 downstream of the reporter gene, thus resembling their location in the genome relative to the *CHRNB4*. Since rs1948 is located in the *CHRNB4* 3′-UTR, a region known to participate in the stability/instability of the mRNA [56], we thought it necessary to study whether or not the risk allele of the rs1948 is involved in the generation of alternative transcripts and how this SNP may modulate the efficiency of post-transcriptional factors, such as selected microRNAs (miRNAs), in the regulation of gene expression.

Our results advance understanding of the regulation of the *CHRNA3/B4* region in two ways. First, experiments demonstrate that alternative *CHRNB4* 3′-UTRs mediate the allelic effects of SNP rs1948 on luciferase expression. Secondly, miR-138, a microRNA known to play a role in the neuroadaptation to drug abuse [57], [58], [59], leads to decreased gene expression when compared to basal conditions, even though this effect was independent of the rs1948.

## Materials and Methods

### Plasmids and Clones

**Figure 1:**
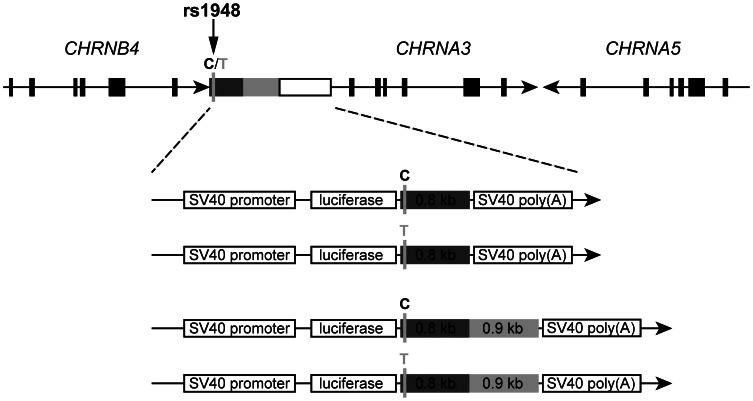
Schematic representation of the CHRNA5/A3/B4 cluster of genes. Schematic representation is shown according to the UCSC Genome Browser Human Feb. 2009 (GRCh37/hg19) Assembly. Arrows indicate direction of transcription. SNP rs1948 is indicated by a vertical arrow in the 3′-UTR of *CHRNB4.* Black boxes on the arrow line represent exons. Different shade of gray in the α3/β4 intergenic region indicates fragments used to design the constructs. The four types of constructs are shown below the cluster, each with the major (black) and minor/risk (grey) allele listed.

Two sequences of different kilobase pairs length (0.8 kb and 1.7 kb), corresponding to fragments downstream of *CHRNB4* and containing the rs1948 SNP, were cloned in separated pGL3-Promoter Luciferase Reporter Vectors (Promega Corporation, Madison, WI, USA). To assess the effect of the rs1948 SNP on gene expression, two pairs (C (major), and T (minor/risk)) each of both sequence length (0.8_C/0.8_T kb, and 1.7_C/1.7_T kb), were synthesized at GenScript, Inc. (Piscataway, NJ, USA) and manipulated in our laboratory to place them downstream of the firefly luciferase gene, thus resembling their location in the genome downstream the *CHRNB4* (−72 kb/+728 kb and −72 kb/+1628 kb, respectively; UCSC:uc002bed.1, see Figure 1). After manipulation, all constructs (pGL3+ insert) were verified by sequencing at SeqWright Inc. (Houston, TX, USA) or GenScript, Inc. (Piscataway, NJ, USA).

### Reagents and Cell Culture

Media was purchased from ATCC (Manassas, VA, USA), unless otherwise indicated. Fetal bovine serum (FBS), and penicillin/streptomycin/amphotericin B (PSA) were purchased from Invitrogen Corporation (Carlsbad, CA, USA). Dibutyryl-cAMP and retinoic acid were obtained from Sigma-Aldrich (St. Louis, MO, USA).

Cell culture conditions have been carried out as described previously by our group [60], [61], [62]. Three neuroblastoma cell lines of different species origin were obtained from ATCC (Manassas, VA, USA), seeded on sterile (gamma irradiated) 75 cm^2^ tissue culture flasks with a negatively charged hydrophilic surface (CELLTREAT Scientific Products, LLC, Shirley, MA, USA), and passaged while in the exponential growth phase. Cells were maintained in a humidified incubator with 5% CO_2_ at 37°C. SH-SY5Y cells (human neuroblastoma) were grown in a 1∶1 mixture of Ham’s F12 (Invitrogen Corporation, Carlsbad, CA, USA) and Eagle’s Minimal Essential Medium (EMEM) with 10% FBS and 1% PSA. Differentiated SH-SY5Y cells (SH-SY5Y-D) were cultured in serum-reduced media with retinoic acid (RA 10 µM, 1% FBS, 1% PSA). B35 (rat neuroblastoma) cells were grown in Dulbecco’s Minimal Essential Medium (DMEM) with 10% FBS and 1% PSA. Neuro2A (mouse neuroblastoma) cells were grown in EMEM with 10% FBS and 1% PSA. Differentiated B35 (B35-D) and N2A cells (N2A-D) were cultured in serum-reduced media with 1 mM Dibutyryl-cAMP (Dibutyryl-cAMP 1 mM, 1% FBS, 1% PSA).

### Cell Transfection and Dual-luciferase Reporter Assay

Cells were seeded in 24-well tissue culture plates (CELLTREAT Scientific Products, LLC, Shirley, MA, USA) at a density of 150,000 cells/mL (48 hour assays) or 100,000 cells/mL (96 hour assays) in a volume of 500 µL/well. Twenty-four hours after seeding, test plasmids (constructs or empty vector) were transfected into cells using 1∶1 ratio of X-tremeGENE HP DNA transfection reagent (µL) (Roche USA, Indianapolis, IN, USA) to test plasmids (µg). Co-transfection with miRNAs (*mir*Vana® miRNA mimic, Invitrogen Corporation, Carlsbad, CA, USA) was performed using 5∶2∶1 ratio of X-tremeGENE siRNA transfection reagent (µL) (Roche USA, Indianapolis, IN, USA), to test plasmids (µg) and miRNA (µg). GIBCO Opti-MEM ® 1 media (Invitrogen Corporation, Carlsbad, CA, USA) was used as serum-free diluent. As a control for transfection, a renilla luciferase plasmid, (pRL-CMV, Promega Corporation, Madison, WI, USA) was co-transfected (0.2 pg/µL) with each construct or empty vector. An empty pGL3 vector and a random sequence miRNA molecule (*mir*Vana™ miRNA mimic, Negative Control #1, Invitrogen Corporation, Carlsbad, CA, USA) that has been validated to produce no identifiable effects on known miRNA function (control miR) were used as control of luciferase expression. Cells were maintained for another 48 hours or 96 hours (undifferentiated and differentiated cells) before harvesting and assaying for luciferase activity. The dual-luciferase reporter assay system (Promega Corporation, Madison, WI, USA) was used to assess gene expression, per manufacturer’s instructions. Two different maxi-preps were tested on at least two different days for each construct as well as for the “empty” pGL3 (no insert).

### RT-PCR

A step-down RT-PCR (HotStarTaq DNA Polymerase, Qiagen, Valencia, CA, USA) protocol was used to determine whether 0.8 kb and 1.7 kb constructs generate the same transcripts. Total RNAs were extracted from N2A cells non-transfected or 48 hours after transfection with pGL3, 0.8_C/T kb, or 1.7_C/T kb constructs using the RNeasy Mini Kit (Qiagen, Valencia, CA, USA). Samples were double treated with DNAses using RNase-Free DNase I Set (Omega bio-tek, Norcross, GA, USA) and RQ1 RNase-Free DNase (Promega, Madison, WI, USA) and then reverse transcribed with High Capacity cDNA Reverse Transcription Kit (Applied Biosystems, Carlsbad, CA, USA). The obtained cDNAs were used as templates. Forward (primer 1, 5'-CCTCATAAAGGCCAAGAAGG-3') and reverse primers (primer 2, 5′-ACCCAGAAAGAAGCAGCAAA-3'; primer 3, 5′-CCATGCCTGAAGCATAGTAGG-3'; primer 4, 5′-GGAGCAAAGAATGGATTGGA-3' and primer 5, 5′-GGGTAGGAGCCATTCATTCA-3') were designed according to the 0.8 kb and 1.7 kb sequences and predicted poly(A) signals (HCpolya). To address whether the RT-PCR products were due to contamination of vector DNA, control reactions using RNA as templates were used (no-RT). Aliquots of the RT-PCR products were resolved on 2% agarose gel, stained with ethidium bromide (Sigma-Aldrich, St. Louis, MO, USA) and visualized on a UV transilluminator.

### 3′-RACE

The 3′-RACE system for rapid amplification of cDNA ends (Invitrogen, Carlsbad, CA, USA) was used to identify the length and number of RNA species generated by each construct (empty pGL3 vector, 0.8 kb and 1.7 kb). Briefly, first strand cDNA synthesis was initiated at the poly(A) tail of 2 µg of total RNA from N2A cells, non-transfected or transfected with empty pGL3, 0.8_C/T kb, or 1.7_C/T kb constructs, using the adapter primer (AP). Specific cDNA was then amplified by PCR (HotStarTaq DNA Polymerase, Qiagen, Valencia, CA, USA) using a gene-specific primer (primer 1, 5′-GCGGTCGGTAAAGTTGTTCC-3') that anneals to a known exon sequence of the luciferase gene, and the Invitrogen kit Abridged Universal Anchor Primer (AUAP). To generate a specific amplification product, a second gene-specific primer was designed to re-amplify luciferase transcripts from the empty pGL3 vector (primer 2, 5′-CCTCATAAAGGCCAAGAAGG-3') and from 0.8_C/T kb, or 1.7_C/T kb constructs (primer 3, 5′-CAGTTCAATTCTGGCCTGTCT-3'). Aliquots of the PCR products were resolved in a Cresyl Violet gel and subsequently purified with Zymoclean Gel DNA Recovery Kit (Zymo Research, Irvine, CA, USA) and sent for sequencing at SeqWright Inc. (Houston, TX, USA) or GenScript, Inc. (Piscataway, NJ, USA).

### Statistical Analysis

Luciferase activity of the test plasmids was divided by luciferase activity of the control of transfection plasmid for each well, yielding a gene expression ratio. Replicate readings were averaged to obtain one ratio value per transfected cell-culture well. Values were then normalized to the average value of the empty pGL3 vector (no insert) within the same cell line and experiment to yield the vector-normalized ratio, or Relative luciferase Activity. Data were analyzed using IBM® SPSS® Statistics v.19.0 (Somers, NY, USA). Data from cells that were not transfected with miRNAs were analyzed using the General Linear Model (GLM) Univariate Analysis, with expression level as the dependent variable and cell line (SH-SY5Y, B35 or Neuro2A), culture-condition (48 hr, 96 hr, 96 hr differentiated), length (0.8 kb or 1.7 kb), and allele (C (major) or T (minor/risk)) as independent variables. Based on results from the all-inclusive analysis, each of the 3 cell lines were analyzed separately with the GLM Univariate Analysis including culture-condition, length and allele.

Values obtained from cells co-transfected with miRNAs were compared to the average value of the control miR (instead of a pGL3 test plasmid) using the General Linear Model (GLM) Univariate Analysis, with expression levels as dependent variable and miRNA (control miR and miR-3157 or miR-138) culture-condition (48 hr, 96 hr, 96 hr differentiated), length (0.8 kb or 1.7 kb), and allele (C (major) or T (minor/risk)) as independent variables.

## Results

### Allelic Effects of rs1948 on Luciferase Expression

**Figure 2:**
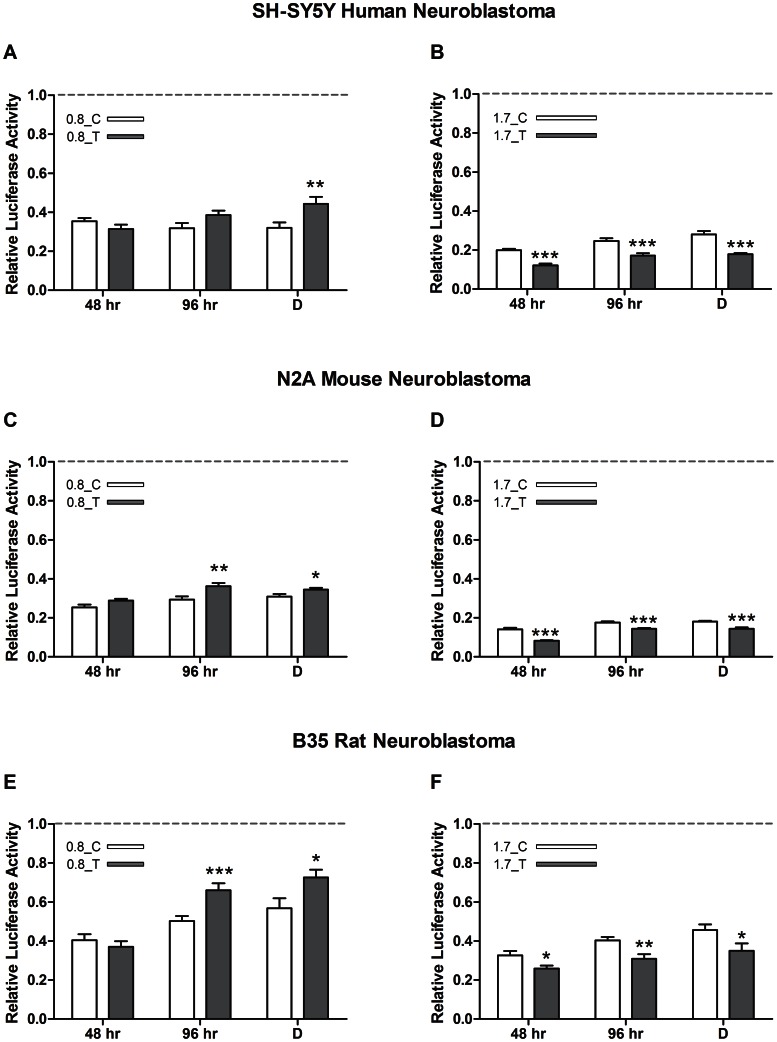
SNP rs1948 modifies luciferase expression. (A, B) SH-SY5Y human neuroblastoma cell line. (C, D) N2A mouse neuroblastoma cell line. (E, F) B35 rat neuroblastoma cell line. Graphs show relative luciferase activity 48, and 98 hr (non-differentiated and differentiated) after transfection with (A, C, E) 0.8 kb or (B, D, F) 1.7 kb constructs. White bars indicate C/major allele. Grey bars indicate T/minor-risk allele. Differences between alleles are indicated with asterisks. Data are shown as mean +/− SEM; ** p<0.01, *** p<0.001. Grey line at 1.0 represents the pGL3 empty vector data.

In order to study whether rs1948 (NC_000015.9) modifies gene expression *in vitro*, a dual-luciferase reporter assay system was performed transfecting different neuroblastoma cell lines of different species origin (human (SH-SY5Y), mouse (N2A) and rat (B35)) with plasmids carrying sequences of different length (empty pGL3 vector, 0.8 kb and 1.7 kb constructs) and tested at different time points or conditions (48 hr, 96 hr (undifferentiated and differentiated cells)). The overall analysis of the data showed a similar allelic effect of rs1948 on luciferase expression across cell lines (F_(2,653)_ = 0.344, ns; ANOVA, Fig. 2). However, this effect was different depending on the length of the construct (F_(1,653)_ = 40.438, p<0.001; ANOVA).

Although the allelic effects were also significantly different between culture-condition (F_(2,653)_ = 3.202, p<0.05; ANOVA), the overall results showed that the minor/risk allele (T) increased luciferase expression compared to the major allele (C) when the rs1948 was in the context of the 0.8 kb sequence (SH-SY5Y, F_(1,74)_ = 5.927, p<0.05; N2A, F_(1,82)_ = 16.742, p<0.001; B35, F_(1,98)_ = 9.579, p<0.01; ANOVA, Fig. 2A, C, E). However, when the rs1948 was studied in the 1.7 kb sequence, the minor/risk allele (T) showed decreased luciferase expression when compared to the major allele (C) (SH-SY5Y, F_(1,69)_ = 83.329, p<0.001; N2A, F_(1,82)_ = 70.182, p<0.001; B35, F_(1,100)_ = 19.744, p<0.001; ANOVA, Fig. 2B, D, F).

### The Use of Alternative CHRNB4 poly(A) Signals Depends on SNP rs1948 and the Length of the Construct

**Figure 3:**
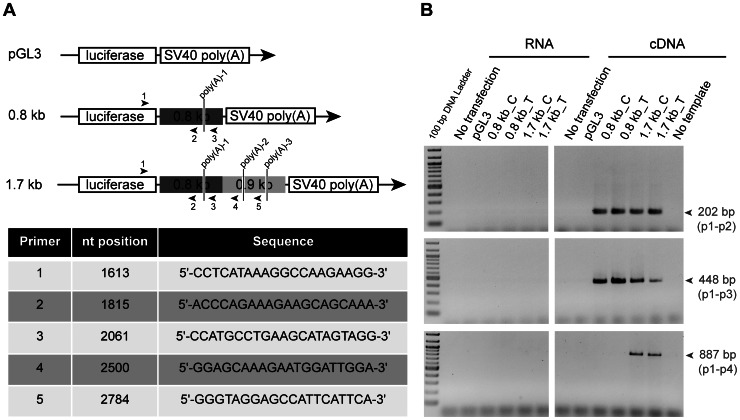
The length of transcripts generated is construct-dependent. (A) Schematic representation of the empty pGL3 vector and 0.8 kb and 1.7 kb constructs. Predicted poly(A) signals are indicated in each construct in between primers used. The numbers and little arrows indicate forward (above the construct) and reverse (below the construct) primers used. The table below shows the nucleotide position of each primer, relative to the transcription initiation site of luciferase gene, and its sequence. (B) PCR products using pairs of primers (1–2), (1–3), and (1–4) and RNA or cDNA templates obtained from N2A cells without transfection or either transfected with empty pGL3 vector or 0.8_C/T and 1.7_C/T kb constructs. RNA was used as template to control for bacterial DNA contamination.

To determine whether the opposite effects of rs1948 observed in the context of the 0.8 kb or the 1.7 kb *CHRNB4* 3′-fragments were due to the generation of alternative transcripts, we performed RT-PCR using pairs of primers limiting predicted poly(A) signals (HCpolya, see Figure 3A). There are three predicted poly(A) signals shown on Fig. 3A, only one of these resides within the 0.8 kb fragment. Primer pair 1–2 was designed to amplify the region immediately upstream of poly(A)-1. Primer pair 1–3 will amplify immediately downstream of poly(A)-1, within the 0.8 kb fragment. Primer pair 1–4 will amplify downstream of poly(A)-1, upstream of poly(A)-2, but outside the 0.8 kb fragment, so it should be specific to the 1.7 kb constructs. Primer 5 is located downstream of poly(A)-2, but upstream of poly(A)-3. Results from PCR showed product p1-p2 (202 bp) amplification from cDNA of N2A cells transfected with either 0.8_C/T kb or 1.7_C/T kb constructs, as expected. In addition, there was a p1-p3 product of 448 bp, suggesting the presence of a poly(A) signal downstream of poly(A)-1 (Fig. 3A, B). With primer pair p1-p4, a product of 887 bp was observed only for the 1.7 kb. There was no product observed for primer pair p1-p5 (data not shown), suggesting that poly(A)-2 or a poly(A) signal near poly(A)-2 is the most likely signal being used for constructs carrying the 1.7 kb fragment.

**Figure 4:**
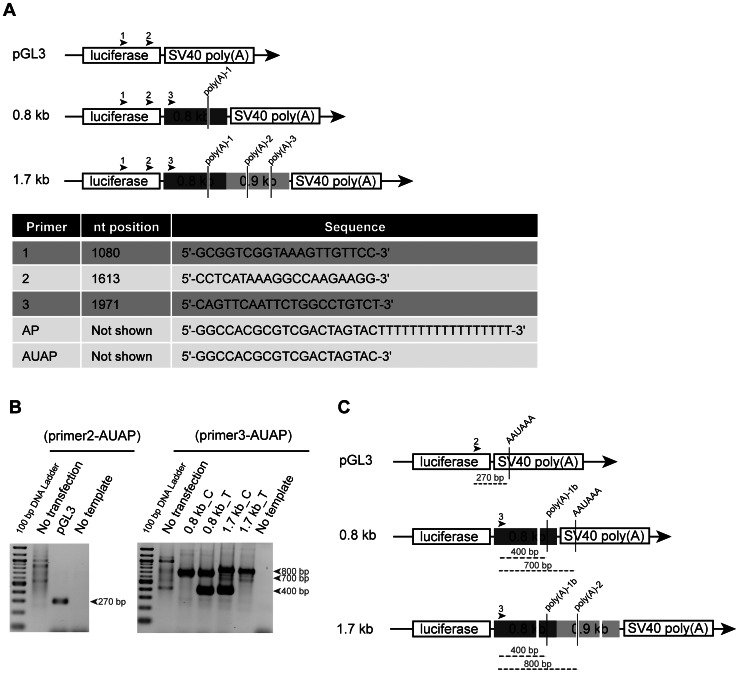
The use of alternative *CHRNB4* poly(A) signals depends on SNP rs1948 and length of construct. (A) Schematic representation of the empty pGL3 vector and 0.8 kb and 1.7 kb constructs. Predicted poly(A) signals are indicated in each construct in between primers used. The numbers above each construct indicate gene-specific primers used. The table below shows the nucleotide position of each primer, relative to the transcription initiation site of luciferase gene, and its sequence. (B) Re-amplified PCR products using pairs of primers (2-AUAP) for pGL3 cDNA templates and (3-AUAP) for 0.8_C/T kb and 1.7_C/T kb constructs. (C) Schematic representation of each construct showing the real poly(A) signal used in each case, determined by the amplification product obtained (dashed grey lines) using pair of primers (2-AUAP) and (3-AUAP).

In order to identify the length and number of RNA species generated by each construct (0.8 kb and 1.7 kb), rapid amplification of cDNA ends (RACE) was performed (Fig. 4) and products obtained were purified and sequenced. cDNA generated from N2A cells transfected with empty pGL3 vector yielded a single product of 270 bp corresponding to a transcript using the SV40 poly(A) signal (Fig. 4B, C). In the construct containing the 0.8 kb fragment, the same SV40 poly(A) signal generated a bigger transcript (700 bp) (Fig. 4B, C). However, a smaller PCR product was also detected with the 0.8_T kb construct, corresponding to a transcript using an unpredicted poly(A) signal (poly(A)-1b) (Fig. 4B, C). This product was also detected with the 1.7_C kb construct. In addition, both 1.7_C and 1.7_T kb constructs generated bigger transcripts (800 bp) that used the predicted poly(A)-2 signal (Fig. 4B, C).

### MiR-3157 and miR-138 Modify the Effects of 0.8 kb and 1.7 kb Constructs on Luciferase Expression

Since the use of alternative *CHRNB4* poly(A) signals depends on SNP rs1948 and the length of the construct, we thought it important to study whether this SNP and/or the length of the construct modulates the efficiency of post-transcriptional factors, such as selected miRNAs, in the regulation of gene expression. Computational analyses from different databases (www.mirbase.org/, http://www.microrna.org/, and http://www.targetscan.org/) predicted miR-3157 (MIMAT0015031) and miR-138 (MIMAT0000430) to bind the 3′-UTR of *CHRNB4* gene, where SNP rs1948 is located. Specifically, the rs1948 is located in the seed sequence of the miR-3157, while miR-138 is predicted to bind a region of the *CHRNB4* 3′-UTR located 48 bp downstream of rs1948. Despite its location, we chose to also test the miR-138 because of its suggested role in the neuroadaptation to drug abuse [57], [58], [59].

**Figure 5:**
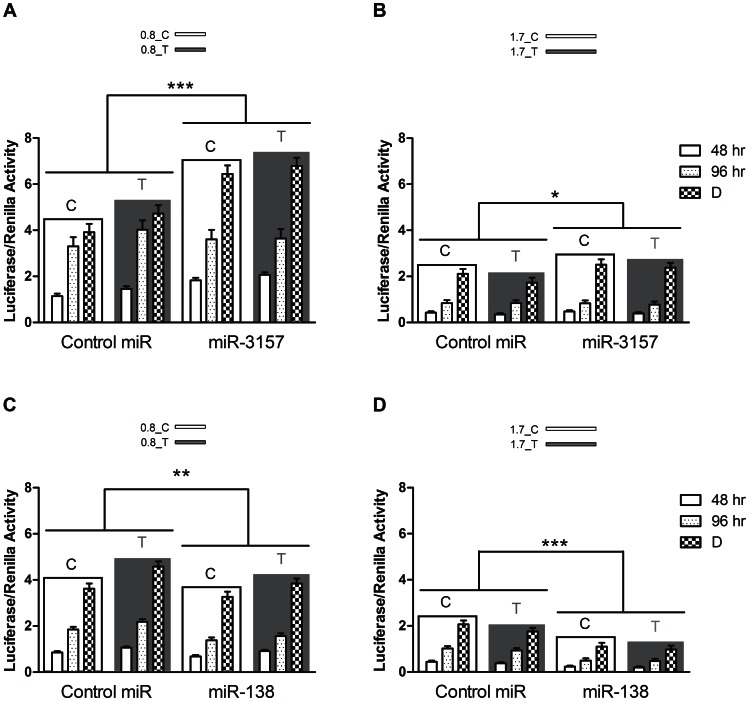
Selected miRNAs modify luciferase expression. (A, B) miR-3157. (C, D) miR-138. Graphs show relative luciferase activity 48, and 98 hr (non-differentiated and differentiated) after co-transfection of pGL3 constructs carrying (A, C) 0.8 kb or (B, D) 1.7 kb inserts, and miRNAs (control miR, miR-3157, and miR-138). Data from different alleles are framed in white (C/major) or grey (T/minor-risk). Asterisks denote differences between the control miR and testing miRNAs (miR-3157 and miR-138). Data are shown as mean +/− SEM; * p<0.05, ** p<0.01, *** p<0.001.

The overall allelic effects on luciferase expression in cells co-transfected with the control miR were not different from those of cells co-transfected either with miR-3157 (F_(1,576)_ = 0.485, ns; ANOVA, Fig. 5A, B) or miR-138 (F_(1,504)_ = 0.182, ns; ANOVA, Fig. 5C, D), thus discarding any possible effect of rs1948 on the efficiency of these miRNAs. The same interaction, allele x length of the construct, previously observed in N2A cells without co-transfection with miRNAs and shown in figure 2C, and D was also found in this experiment when cells were co-transfected with control miR (F_(1,360)_ = 8.745, p<0.01; ANOVA, Fig. 5), thus confirming the results obtained before.

Although no interaction between the allelic effects of rs1948 on luciferase expression and the effects of miR-3157 and/or miR-138 were found, there was a main effect of both miRNAs; miR-3157 increased the overall luciferase expression (F_(1,576)_ = 35.515, p<0.01; ANOVA, Fig. 5A, B) while miR-138 decreased it (F_(1,504)_ = 55.522, p<0.001; ANOVA, Fig. 5C, D) when compared to that observed with the control miR. The increased luciferase expression observed with miR-3157 was dependent on the length of the construct, with a more pronounced effect on constructs carrying the 0.8 kb sequence compared to cells co-transfected with constructs carrying the 1.7 kb sequence (F_(1,576)_ = 18.582, p<0.001; ANOVA, Fig. 5A, B). In contrast, the decreased luciferase expression caused by miR-138 was greater when cells were co-transfected with constructs carrying the 1.7 kb sequence (F_(1,504)_ = 6.217, p = 0.01; ANOVA, Fig. 5C, D).

## Discussion

A previous study from our laboratory showed an association between SNP rs1948, a genetic variant located in the *CHRNB4* 3′-UTR, and early age of initiation to nicotine and alcohol use [30]. Herein, we demonstrate that SNP rs1948 alters luciferase expression when sequences carrying this genetic variant were cloned downstream of the luciferase gene, a re-creation of rs1948 in relation to the *CHRNB4* gene.

Several studies, looking at the transcriptional level, have revealed a cell-type specific enhancer positioned in the rat β4 3′-UTR [37], [39], [42], [44], [48], [50]. By contrast, we found decreased luciferase expression in all our test plasmids when compared to the empty pGL3 vector, along with no differences among cell lines tested. Although our results differ from those studies, it could be because in the present study we cloned fragments located downstream of the human β4-nicotinic receptor subunit rather than those from rat. Even though *CHRNB4* is located in a cluster of genes, conserved throughout vertebrates [63], [64], [65], it is known that 3′-UTRs are less conserved across species than protein-coding sequences [66], [67]. Indeed, the 3′-UTR of this gene appears to be poorly conserved between rat and human as noticed when both regions are aligned (Fig. S1). In particular, the 187 bp β4 3′-UTR rat fragment, reported by McDonough and colleagues in 1997 to exhibit an enhancer activity, does not align with the human β4 3′-UTR, therefore suggesting the participation of other regulatory elements.

Since 3′-UTRs play a pivotal role in modulating mRNA stability [56], and translation [68], [69]; we focused our study on the post-transcriptional regulation of the β4-nicotinic receptor subunit. Interestingly, we found that the effects of rs1948 on gene expression were opposite depending on the length of the fragments cloned (0.8 kb or 1.7 kb). The main post-transcriptional factors that influence regulation of gene expression are microRNAs which bind the 3′-UTR of their target mRNAs and induce their degradation and/or prevent their translation [70], [71]. Given that mRNAs with shortened 3′-UTRs are likely to escape this type of negative regulation, as demonstrated in proliferating [72] and tumor cells [73], [74], we thought it necessary to ascertain whether the opposite effects of rs1948 observed from constructs of different length were due to the length of 3′-UTRs from mRNAs generated by the 0.8 kb and 1.7 kb constructs. Our results demonstrate that 0.8 kb and 1.7 kb constructs generate transcripts of different length, thus suggesting that alternative cleavage and polyadenylation depend on the length of the fragment cloned. Our data from 3′-RACE system for rapid amplification of cDNA ends and subsequent sequencing corroborates the hypothesis that these constructs use different poly(A) signals, therefore generating mRNAs with different 3′-UTR lengths.

Interestingly, we also found differences in the number and length of mRNA species generated between alleles, which is consistent with the different rs1948 effects observed in luciferase expression. These results showed that 0.8_T kb and 1.7_C kb constructs generated an additional transcript shorter than that in the 0.8_C kb and 1.7_T kb constructs. Although this finding is surprising and counterintuitive, it is known that mRNAs form secondary and tertiary structures and that alteration in these structures represent a well-known regulatory mechanism for many RNA cellular processes [75]. Thus, it is not surprising that constructs of different length might undergo alternative cleavage and polyadenylation processes because of their different structural folds and generate transcripts with different 3′-UTRs length. It is also known that a SNP can lead to different structural folds of mRNA that subsequently can affect its stability [76] or protein-RNA interactions associated to the polyadenilation process [77], [78]. This may be an explanation of why we found differences in the use of different polyadenylation sites between constructs of different rs1948 alleles. Another possibility could be that the SNP is affecting a specific sequence for an RNA-binding regulatory factor whose function is recruiting deadenylases. In this case, the SNP would increase or decrease the rate of poly(A) tail degradation [79], depending on the allele, and subsequent RNA decay. In our experiments, luciferase expression of 0.8_T kb was higher than that of 0.8_C kb, and 1.7_C kb higher than that of 1.7_T kb, so these results reinforce the idea that shortened 3′-UTRs are more stable, perhaps because of their fewer miRNA binding sites. Although the allelic effects on luciferase expression are opposite depending on the surrounding sequence (0.8 kb or 1.7 kb) we consider the 1.7 kb constructs more physiological relevant since these constructs used internal poly(A) signals, while the 0.8 kb constructs used the SV40 poly(A) signal, present in the luciferase vector, to generate one of the transcripts. In this regard, the decreased luciferase expression found with the risk allele (T) in the 1.7 kb context, and previously associated with early age of initiation to nicotine and alcohol use by Schlaepfer and collegues, correlates with the study of Frahm et al., 2011 in which mice overexpressing the β4-nicotinic receptor subunit showed increased aversive effects of nicotine. However, data obtained from the 0.8 kb constructs are also relevant since it demonstrates that luciferase expression directly depends on the 3′-UTR length of transcripts generated.

In the luciferase experiments, co-transfecting these constructs with selected miRNAs showed a differential effect based on the length of the construct, but this effect was independent of the rs1948 allele. Surprisingly, co-transfection of 0.8 kb and 1.7 kb constructs with miRNA-3157 increased the overall luciferase expression. However, since miRNAs are usually known to negatively regulate the expression of their targeted mRNAs, it is possible that this miRNA is decreasing the expression of other genes that could be negatively modulating the expression of luciferase. More important is the finding that miR-138 decreased luciferase expression when co-transfected with both 0.8 kb and 1.7 kb constructs. This observation suggests that miR-138 could be important in the regulation of β4-nicotinic receptor subunit expression and contribute the susceptibility to nicotine and alcohol addiction. A limitation of the study is the fact that we cannot directly demonstrate binding of miR-138 to the seed sequence in the 3′-UTR. One possibility would be to abolish the seed sequence, but as with most miRNAs, miR-138 is likely to affect many genes, which in turn affect regulation of *CHRNB4*, and be the initial source of the differences in expression we observed.

It has been demonstrated that overexpressing the *Chrnb4* gene in the mouse increases the aversive effects of nicotine [35]. Thus, it is important to identify factors involved in the regulation of this gene, which has also been associated with addictive behaviors in humans [30], [80], [81]. In this study we provide evidence for two mechanisms related to the 3′-UTR of the human genes. The first is an allelic effect of SNP rs1948 where the risk allele (T), associated with early of initiation to nicotine and alcohol use [30], leads to decreased luciferase expression in the 1.7 kb context (the most physiological relevant). Secondly, there are independent functional effects on expression resulting from the presence of two miRNA binding sites in the 3′-UTR.

In conclusion, our results show that rs1948 modifies gene expression and that this effect seems to be mediated by the fact the *CHRNB4* generates mRNAs with different 3′-UTR lengths. Moreover, this is the first report of a post-transcriptional regulation of this subunit by miR-138, thus identifying a potential target for the treatment of nicotine and alcohol addiction.

## Supporting Information

Figure S1 **Rat-Human** ***Chrnb4*** **3′-UTR similarities.** (A) Schematic representation of the 2800 bp Rat *Chrnb4* 3′-UTR. Grey boxes indicate nucleotide similarities with the 1700 bp Human *Chrnb4* 3′-UTR. The Rat 187 bp fragment reported by McDonough and colleagues to exhibit an enhancer activity is shown in the diagram between 484 bp and 671 bp downstream of *Chrnb4*. (B) Alignment of the Rat and Human *Chrnb4* 3′-UTR sequences according to the Nucleotide Blast from the National Center for Biotechnology Information (NCBI) when settings were set up at “somewhat similar sequences (blastn)”. Notice that the 187 bp (484 bp-671 bp) Rat *Chrnb4* 3′-UTR fragment does not align with the human *Chrnb4* 3′-UTR.
(TIF)
