# Supplementary figures and images for: Alternative CHRNB4 3′-UTRs Mediate the Allelic Effects of SNP rs1948 on Gene Expression
Source: PLoS One. 2013 May 14;8(5):e63699. doi: 10.1371/journal.pone.0063699 (PMC3653846; doi:10.1371/journal.pone.0063699)

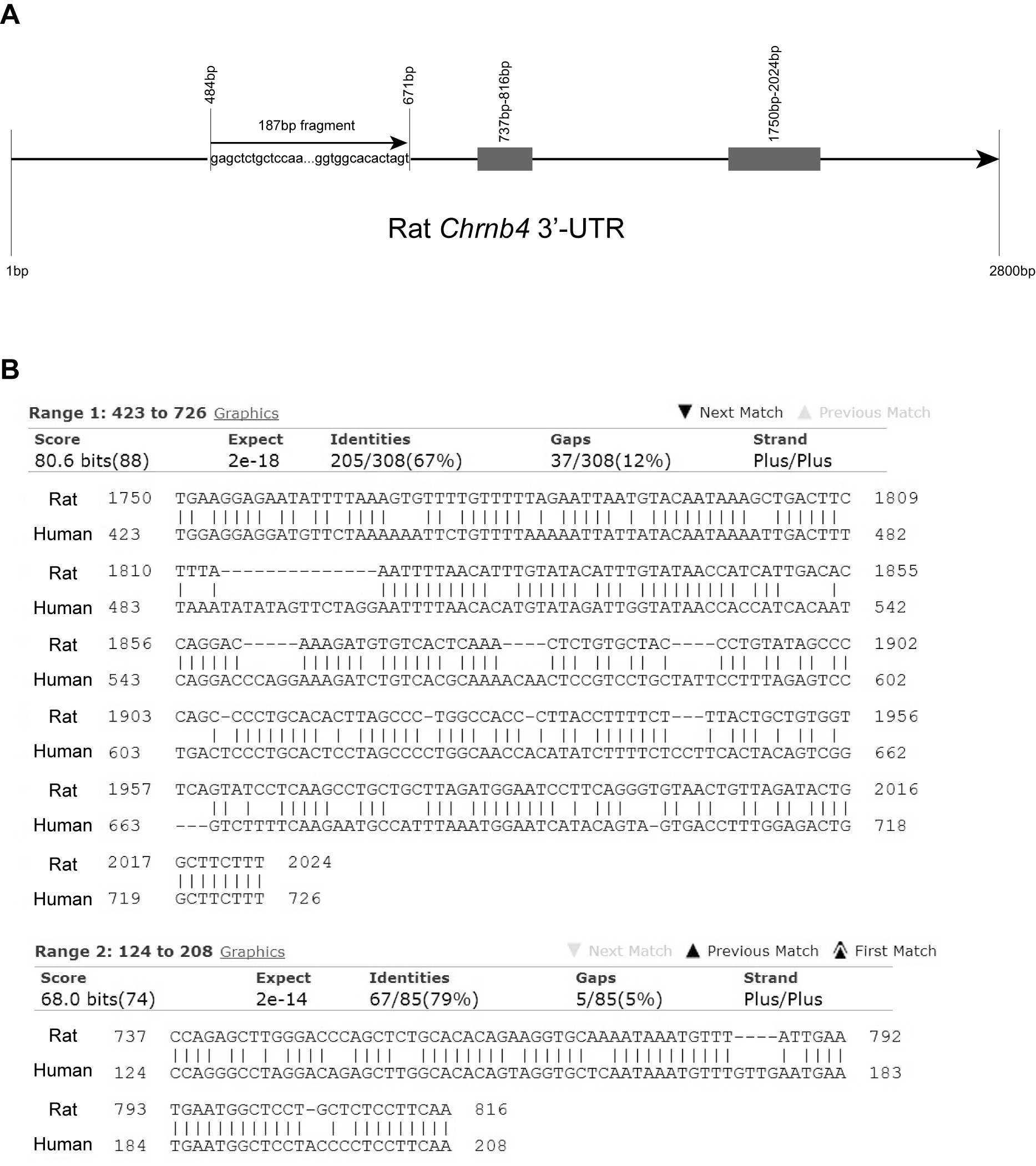

Supplement: Figure S1 — Rat-Human Chrnb4 3′-UTR similarities. (A) Schematic representation of the 2800 bp Rat Chrnb4 3′-UTR. Grey boxes indicate nucleotide similarities with the 1700 bp Human Chrnb4 3′-UTR. The Rat 187 bp fragment reported by McDonough and colleagues to exhibit an enhancer activity is shown in the diagram between 484 bp and 671 bp downstream of Chrnb4. (B) Alignment of the Rat and Human Chrnb4 3′-UTR sequences according to the Nucleotide Blast from the National Center for Biotechnology Information (NCBI) when settings were set up at “somewhat similar sequences (blastn)”. Notice that the 187 bp (484 bp-671 bp) Rat Chrnb4 3′-UTR fragment does not align with the human Chrnb4 3′-UTR. (TIF) [file pone.0063699.s001.tif]
